# Aerobic exercise combined with memory strategy training improve the cognitive function

**DOI:** 10.1002/brb3.3234

**Published:** 2023-08-23

**Authors:** Ning Zhao, Xiuxia Yao, Yafei Wang, Xiao Chen, Zhaoxia Wang

**Affiliations:** ^1^ Department of Nursing Cangzhou Central Hospital Cangzhou Hebei China

**Keywords:** aerobic exercise, cognitive impairment, memory strategy training, type 2 diabetes mellitus

## Abstract

**Background:**

Type 2 diabetes mellitus (T2DM) is closely associated with the occurrence of cognitive impairment, imposing a heavy burden on the patient's family and society. Aerobic exercise and targeted memory strategies have been widely reported to improve cognitive function.

Methods: A total of 122 T2DM patients with Montreal Cognitive Assessment Scale (MoCA) test scores of less than 26 received the aerobic exercise combined with memory strategy training. After 6 months of intervention, a final group of 113 patients entered the final evaluation and analysis. Diabetes‐specific quality of life scale (DSQL) and activities of daily living (ADL) assessments were performed to evaluate the life quality of the patients.

**Results:**

The scores of MoCA and ADL were significantly upregulated, and the scores of DSQL were significantly reduced after the 6‐month intervention of T2DM patients. The levels of fasting plasma glucose (FPG), hemoglobin A1c, total cholesterol (TC), and triglyceride (TG) levels of T2DM patients with cognitive impairment significantly decreased post intervention. A significant decrease in low density lipoprotein cholesterol (LDL‐C) and an increase in high density lipoprotein cholesterol (HDL‐C) were observed. The FPG, HbA1, TC, TG, and LDL‐C levels were significantly lower, and the HDL‐C levels were significantly higher in patients with normal cognitive function than in patients with abnormal cognitive function.

**Conclusions:**

Aerobic exercise combined with memory strategy training effectively improved the memory and cognitive function in patients with T2DM.

## Graphical abstract

1. The levels of fasting plasma glucose (FPG), hemoglobin A1c (HbA1c), total cholesterol (TG) and triglyceride (TG) levels of T2DM patients with cognitive impairment significantly decreased post intervention. 2. Aerobic exercise combined with memory strategy training effectively improved the memory function and cognitive function in patients.

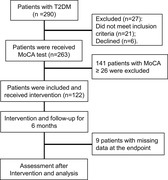

## 1 INTRODUCTION

Diabetes mellitus (DM) is a group of noninfectious, systemic metabolic diseases characterized by chronic hyperglycemia caused by a combination of genetic and environmental factors, of which about 90% are type 2 DM (T2DM) (Bailes, 2002). T2DM is closely related to the occurrence of cognitive dysfunction (Srikanth et al., 2020). As the disease progresses, patients may show learning disabilities and memory loss, and severe cases may develop dementia, which imposes a heavy burden on the patient's family and society (van Sloten et al., 2020). Sustained hyperglycemia in patients could cause damage to the cerebral nerves, resulting in impaired cognitive function in the brain (Lyu et al., 2020). Dementia might be caused when the disease continues to progress, affecting the ability to perform activities of daily living (ADL) and reducing the quality of life of patients (Moran et al., 2019). In the past, the main principles of medical care for T2DM patients were blood sugar compliance and control (Tan et al., 2019). There was no emphasis on improving the ADL and cognitive dysfunction of patients, and targeted strategies should be selected to promote the significant improvement of cognitive function in patients with T2DM.

Aerobic exercise is one of the most commonly used treatment methods in T2DM rehabilitation intervention (Kirwan et al., 2017). There are various types of aerobic exercise therapy, and common sports include walking, cycling, jogging, tai chi, swimming, aerobics, and fitness dance (Wang et al., 2022). The relevant meta‐analysis results show that aerobic exercise could improve the memory ability and executive ability in cognitive function (Sampath Kumar et al., 2019). Memory strategy training promotes the information processing process of patients mainly through cognition, maintenance, recognition, or recall of past events, so that the memory ability of patients could be effectively maintained (Poblete‐Aro et al., 2018). Interval retrieval method and error‐free learning method are commonly used memory training strategies in elderly patients, which could effectively improve the performance of patients in memory tests and improve the memory level of patients (McEwen et al., 2018). As a visual stimulus, pictures could effectively feedback intuitive facial information and allow patients to understand semantic information, such as gender, age, and name (Sahadevan et al., 2021). Reading picture stories and watching illustrated scenes could allow patients to develop their thinking and actively acquire relevant knowledge (Sahadevan et al., 2021). Diary writing may involve motor sense, hearing, vision, and so forth, and also needs to coordinate various cognitive processes such as memory, comprehension, logic, language, and thinking, which could assist in the training and intervention of memory function (von Bastian & Oberauer, 2014). The Montreal Cognitive Assessment (MoCA) assessment is highly sensitive to the detection of mild cognitive impairment (Ciesielska et al., 2016). The MoCA assessment involves many areas of cognitive function, including memory, attention, abstract ability, visuospatial and executive function, and orientation (Ciesielska et al., 2016). Our hospital has carried out aerobic exercise combined with memory strategy training to provide nursing intervention for T2DM patients with cognitive impairment for many years. Therefore, the present study conducted a retrospective analysis on the improvement of cognitive dysfunction and the improvement of glucose and lipid metabolism in T2DM patients who underwent aerobic exercise combined with memory strategy training.

## 2 METHODS

### 2.1 Participants

**FIGURE 1:**
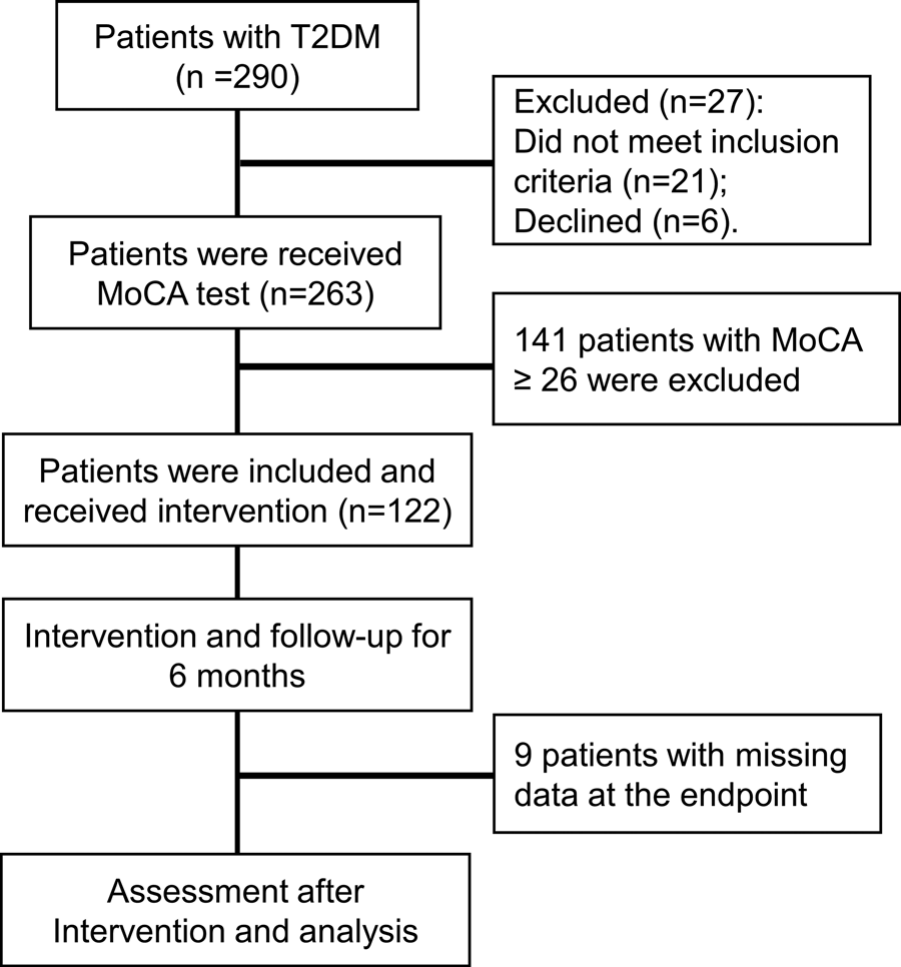
The flowchart diagram of this study.

To investigate the effect of aerobic exercise combined with memory strategy training on cognitive function in patients with type two diabetes and cognitive impairment, a retrospective study was performed. The flowchart diagram of this study is shown in Figure 1. Of the 290 screened T2DM patients, 263 underwent our MoCA test. Among the 263 T2DM patients, 122 had MoCA scores of less than 26. These 122 patients received aerobic exercise combined with memory strategy training. After 6 months of intervention, 9 patients were lost and 113 patients entered the final evaluation and analysis.

Inclusion criteria: (1) The patients were diagnosed with T2DM by the “Chinese Guidelines for the Prevention and Treatment of Type 2 Diabetes (2013 Edition)”; (2) age ≥60 years; (3) years of education ≥6 years; (4) no recent use of drugs that affect cognitive function; (5) normal audiovisual function; (6) MoCA total score <26 points.

Exclusion criteria: Patients with (1) severe complications of diabetes; (2) history of severe neurological or psychiatric diseases, such as dementia, Parkinson's disease, and stroke; (3) anxiety and depression; (4) history of alcohol or drug abuse; (5) history of cardiovascular disease (heart insufficiency, severe arrhythmia, heart failure, and myocardial infarction); (6) severe liver and kidney insufficiency; or (7) malignant tumors. The study was approved by the ethics committee of Cangzhou Central Hospital. Informed consent was obtained from each participant.

### 2.2 Montreal Cognitive Assessment Scale (MoCA)

MoCA was used in this study to assess patients’ cognitive function before and after the start of care. MoCA is a test used by health care providers to evaluate people with memory loss or other symptoms of cognitive decline. It can help identify those at risk for developing Alzheimer's disease and other forms of dementia. It is also used as a screening tool for conditions like Parkinson's disease, brain tumors, substance abuse, and head trauma. The MoCA used in this study contains 30 questions and takes around 10–12 min to complete. MoCA includes a total score of 30 in cognitive domains, such as attention and concentration, memory, executive function, visual‐spatial skills, language, abstract thinking, calculation, and orientation.

a Visuospatial/executive functioning:

The individual was asked to draw a line connecting numbered circles in ascending order. The individual was asked to copy a three‐dimensional cube.

b Naming:

The examiner showed the individual a line drawing of different animals and asked them to name each one.

c Memory:

The examiner read a list of five words at a rate of one word per second. After the examiner finished reading, the individual was asked to recall as many words as possible.

The examiner read a second list of five words, and the individual was asked to recall these as well. In a later section, the individual was asked to recall the previously presented words.

d Attention:

The individual was read a series of letters and was asked to tap their hand whenever they heard the letter “A.”

e Language:

The individual was asked to repeat two sentences after the examiner. The individual was asked to name as many words as possible that begin with a specific letter within 1 min.

f Abstraction:

The examiner asked the individual to explain the similarity between two objects (e.g., train and bicycle).

g Delayed recall:

The individual was asked to recall the five words from the earlier memory task.

h Orientation:

The individual was asked questions related to the current date, month, year, day, place, and city.

Each task within the MoCA had specific scoring criteria outlined in the test booklet. The examiner assigned points for correct responses, and the total score was calculated by summing up the points for each section. The maximum score was 30 points, with a score of 26 or above typically considered normal. A lower score on the MoCA suggested cognitive impairment or potential cognitive decline. However, a low score alone was not sufficient for diagnosis, and further evaluation was typically required.

### 2.3 Aerobic exercise

Before beginning formal exercise intervention, patients underwent an initial assessment of their motor function and cardiorespiratory fitness. They then received 1 week of adaptive training, during which the exercise intensity was gradually increased and adjusted according to each patient's individual conditions. Our experienced physicians assessed the patient's aerobic exercise requirements daily, based on their gradual increase in physical activity. The exercise criteria for our study incorporated both the physician's experience and the patient's actual perception and tolerance. After a week of acclimatization, patients began formal training. During training, the exercise intensity was monitored by measuring the heart rate and subjectively feeling fatigue level. The intensity was controlled at a moderate level, meaning that the patient felt that the exercise intensity was in the range of “relaxed” to “somewhat strenuous.” The first 5–10 min of each session were dedicated to warm‐up exercises, such as low‐intensity manual exercises or slow walking training. The main items during the formal training phase were brisk walking and jogging. Patients were required to wear a heart rate monitor throughout the entire exercise process, with their heart rate controlled at 50%–70% of their maximum heart rate (220‐age). The target intensity exercise time was 30 min. At the end of each session, patients received 5–10 min of restorative exercise, such as slow walking or manual exercises. The exercise start time was selected to be 1 h after dinner. Patients were instructed to carry a card with their name, disease, and family contact information recorded during exercise. They were also asked to bring a certain amount of candy, biscuits, and other foods in case they experienced symptoms of hypoglycemia, such as palpitation, fatigue, cold sweat, and hunger during training. In such cases, they were instructed to eat immediately. The training frequency was four times a week (with the interval between exercises recommended not to exceed 1 day), and each aerobic workout lasted 40–60 min. The implementation and supervision of exercise intervention for patients in the aerobic exercise group were the responsibility of the project team's monitoring personnel. These personnel contacted patients every week to inquire about their implementation of the exercise prescription and asked them to provide their exercise diary for the monitoring period. Data was collected in a timely manner. The therapist made reasonable adjustments to the training plan according to each patient's recovery during the training process, so it was slightly adjusted during the study.

### 2.4 Memory strategy training

Memory strategy training was conducted in two parts: (1) training group formation: the patient's attending doctor, responsible nurse, and head nurse formed a memory training guidance group. Group members were required to actively participate in professional memory training and develop a memory training plan after completing the training. During the training process, members needed to make reasonable adjustments and provide targeted guidance according to the patient's recovery. (2) Implementation of targeted memory strategies:

Picture story reading: Picture stories with fewer than 100 words were used as reading training materials. The team members extracted 10 keywords from the location, time, and characters of the story and briefly introduced it to the patient. After viewing the pictures, patients were asked skillful questions by the training group members to guide them in actively thinking about the development of the story and memorizing the 10 keywords. After completing the reading, patients were asked to read the material aloud twice and then retell or try to recall the content of the story. Team members needed to encourage and remind patients if they made mistakes while retelling the story. The above steps were repeated once every 3 min until the patient spoke all the keywords correctly, with the interval reasonably extended to 30 min/time before repeated training.

Face‐name association: Memory training was conducted using 10 photos. Researchers used visual stimulation of facial features to help patients grasp relevant information, such as address, name, and face. After 3 min, patients were retrained. If they answered incorrectly, their mistakes were corrected using an error‐free learning method, and they were required to repeat it once. After 3 min of training, patients were required to repeat it again, with the above steps repeated every 3 min using an interval extraction method. Once patients could accurately memorize all information, the interval time was doubled and training was repeated several times, with each session lasting about 30 min. The above training frequency was four times per week.

Writing a diary: Diary content mainly included people, places, times, and dates involved in memory training, and patients were required to read their diary entries aloud. If information was recorded incorrectly, patients were encouraged to recall the situation and make corrections. Patients were asked to read their diary before going to bed and before meals until they developed a habit of actively reading it.

Aerobic exercise was supervised by professional staff who urged patients to perform it daily at home. Picture story reading and face‐name association were conducted in the hospital with a therapist's assistance at a frequency of four times a week. Writing diaries was done at home once a day without additional assistance. Aerobic exercise and memory training were conducted on the same day. The therapist made reasonable adjustments according to each patient's recovery during the training process, so the training plan was slightly adjusted during the study. The intervention period lasted for 6 months.

### 2.5 Diabetes‐specific quality of life scale (DSQL)

Diabetes‐specific quality of life scale (DSQL) included 27 items in 4 dimensions: physiological function, psychological state, social relationship, and treatment. Each item was scored on a five‐level scale. The higher the score, the lower the quality of life.

### 2.6 Activities of daily living (ADL)

The ADL scale was used to assess the quality of life. The maximum ADL score was 100, where a higher ADL score indicated higher quality of life.

### 2.7 Statistical methods

Prism 9.3 (GraphPad) was used for data analysis. The Anderson–Darling test, the D'Agostino and Pearson test, the Shapiro–Wilk test, and the Kolmogorov–Smirnov test were used to assess the normality of the data before analysis. To compare the MoCA, DSQL, and ADL scores before and after the 6‐month intervention, Wilcoxon signed‐rank test was used. Mann–Whitney *U* test was utilized to analyze the changes in glucose and lipid metabolism between patients with normal cognitive function and those with abnormal cognitive function. The data was presented in mean ± SD or *n* (%).

## 3 RESULTS

### 3.1 Clinical characteristics of the study participants

Baseline characteristics, including age, gender, body mass index, blood pressure, fasting plasma glucose (FPG), hemoglobin A1c (HbA1c), high density lipoprotein cholesterol (HDL‐C), low density lipoprotein cholesterol (LDL‐C), total cholesterol (TC), and triglyceride (TG) levels of the T2DM patients with cognitive impairment, were first collected and shown in Table 1.

**TABLE 1:** Baseline characteristics of the type 2 diabetes mellitus (T2DM) patients with cognitive impairment (*n* = 113).

Age (years)	67.25 ± 7.18
Male, (*n*, %)	65 (57.52%)
T2DM course (years)	8.32 ± 2.59
BMI (kg/cm^2^)	24.17 ± 3.34
Systolic blood pressure (mmHg)	123.64 ± 13.28
Diastolic blood pressure (mmHg)	78.91 ± 9.53
FPG (mmol/L)	8.38 ± 1.48
HbA1c (%)	8.19 ± 1.10
HDL‐C (mmol/L)	1.17 ± 0.31
LDL‐C (mmol/L)	3.12 ± 0.89
TC (mmol/L)	5.18 ± 0.59
TG (mmol/L)	1.81 ± 0.43

*Note*: Values were expressed as *n* (%) or mean expr

Abbreviations: BMI, body mass index; FPG, fasting plasma glucose; HbA1c, hemoglobin A1c; HDL‐C, high density lipoprotein cholesterol; LDL‐C, low density lipoprotein cholesterol; TC, total cholesterol; TG, triglyceride.

### 3.2 Cognitive function and quality of life of patients before and after intervention

**FIGURE 2:**
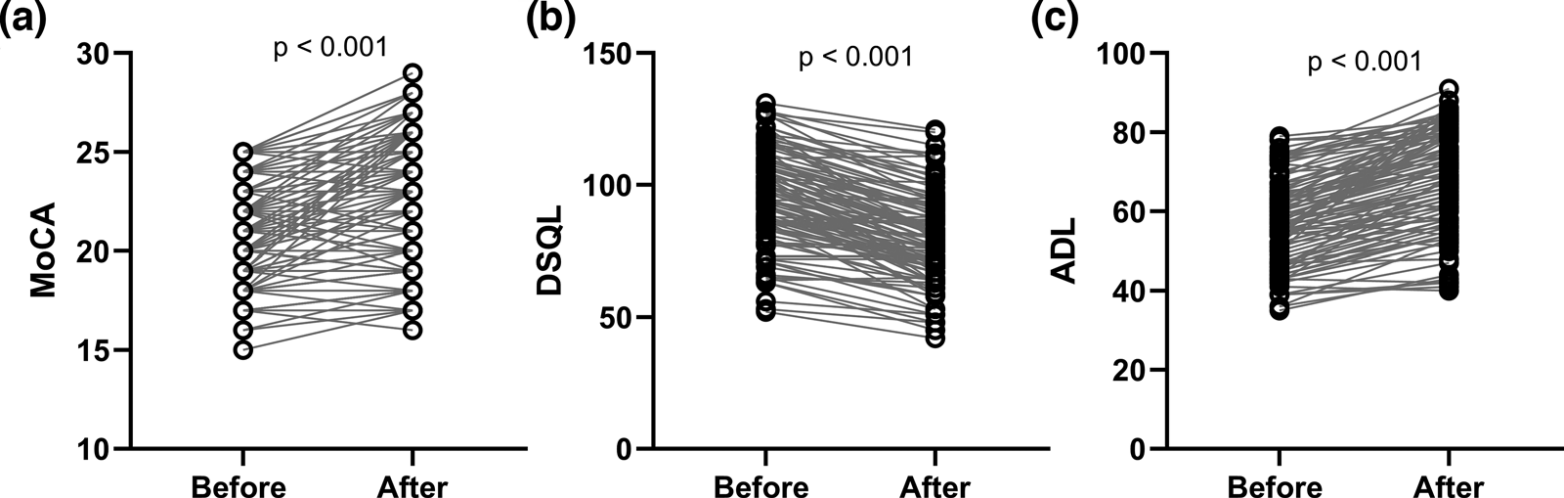
Effects of aerobic exercise combined with memory strategy training for 6 months on Montreal Cognitive Assessment (MoCA) (a), diabetes‐specific quality of life scale (DSQL) (b), and activities of daily living (ADL) (c) in type 2 diabetes mellitus (T2DM) patients with cognitive impairment. *p* Values were calculated from Wilcoxon matched‐pairs signed rank test.

To analyze changes in cognitive function and quality of life in patients before and after our 6‐month intervention, we collected and analyzed MoCA, DSQL, and ADL scores before and after the nursing. As shown in Figure 2a, the scores of MoCA in the patients significantly upregulated after the 6‐month intervention, indicating the improvement of the cognitive functions of the patients (mean value 95.94 vs. 79.57, *p* < .001). Furthermore, the alteration of DSQL (mean value 57.36 vs. 68.97, *p* < .001) and ADL (*p* < .001) scores consistently demonstrated the improvement of diabetic patients after our nursing intervention (Figure 2b,c).

### 3.3 Changes in glycolipid metabolism of patients before and after intervention

**FIGURE 3:**
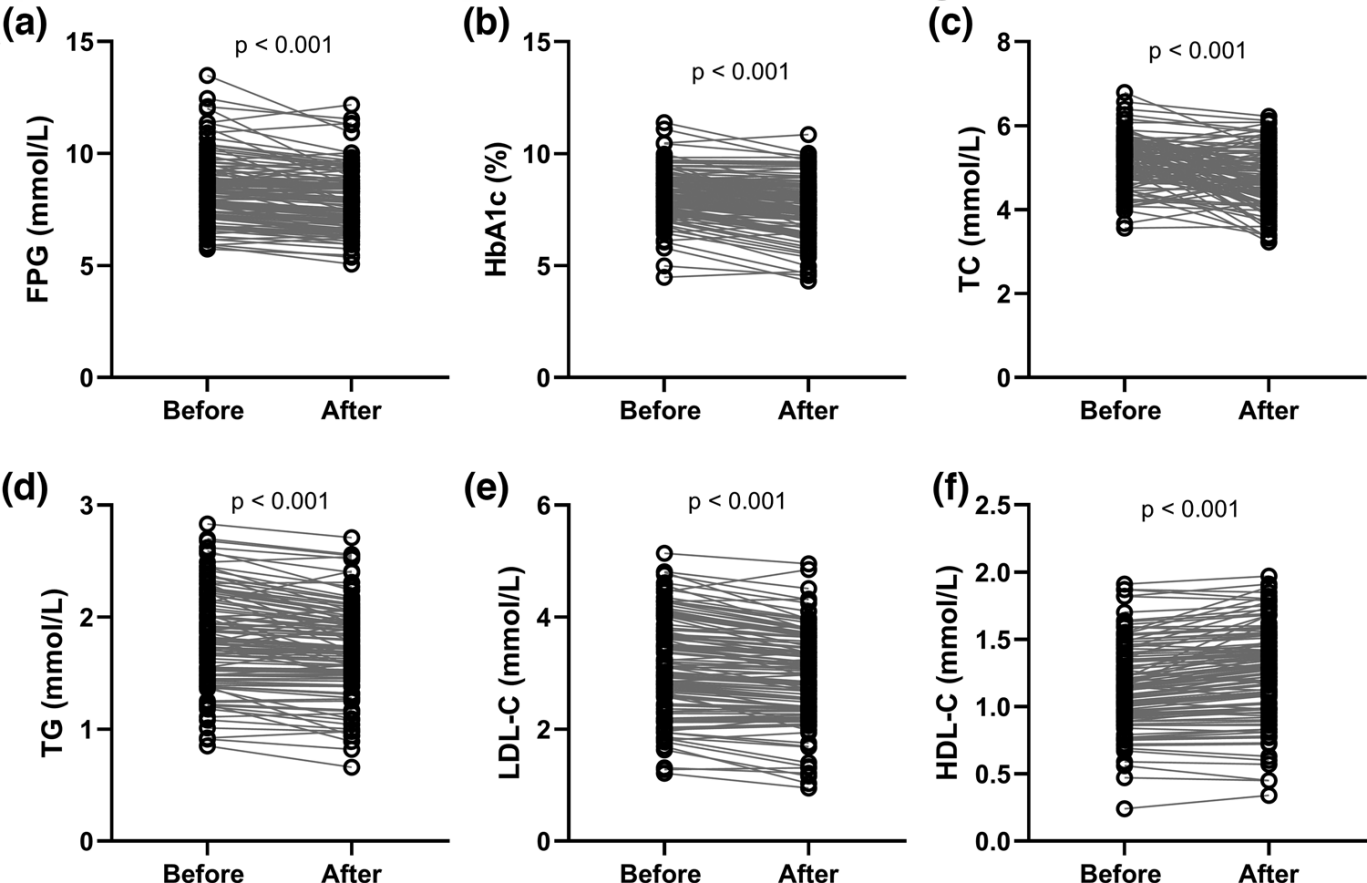
Effects of aerobic exercise combined with memory strategy training for 6 months on fasting plasma glucose (a), hemoglobin A1c (b), total cholesterol (c), triglyceride (d), low density lipoprotein cholesterol (e), and high density lipoprotein cholesterol (f) in type 2 diabetes mellitus (T2DM) patients with cognitive impairment. *p* Values were calculated from Wilcoxon matched‐pairs signed rank test.

To explore the effect of our study on the glucose and lipid metabolism, we analyzed the FPG, HbA1c, TC, TG, LDL‐C, and HDL‐C before and after the intervention in T2DM patients with cognitive impairment. As shown in Figure 3a,b, the FPG levels (mean value 8.377 vs. 7.810, *p* < .001) and HbA1c levels (mean value 8.187 vs. 7.607, *p* < .001) of T2DM patients with cognitive impairment significantly decreased post our intervention. Consistently, the TC levels (mean value 5.177 vs. 4.735, *p* < .001) and TG levels (mean value 1.810 vs. 1.717, *p* < .001) of T2DM with cognitive impairment also significantly decreased post our intervention (Figure 3c,d). In terms of lipid metabolism, our care resulted in a significant decrease in LDL‐C (mean value 3.120 vs. 2.918, *p* < .001) and an increase in HDL‐C (mean value 1.170 vs. 1.234, *p* < .001) among the participants in this study (Figure 3e,f).

### 3.4 Changes of glucose and lipid metabolism before and after intervention in patients with different cognitive function levels

**FIGURE 4:**
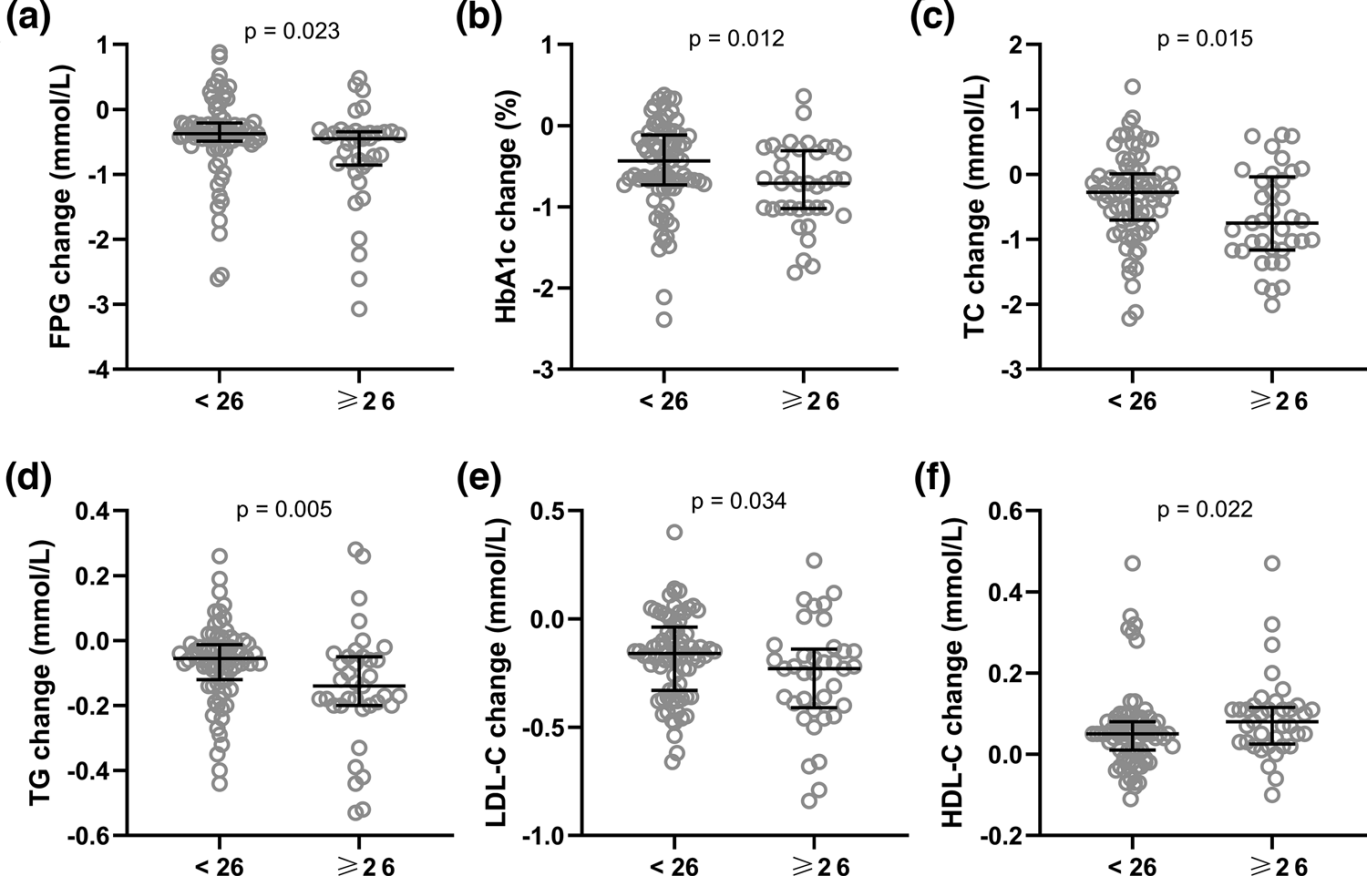
Among 113 type 2 diabetes mellitus (T2DM) patients with cognitive impairment (Montreal Cognitive Assessment [MoCA] < 26), 37 cases become normal after aerobic exercise combined with memory strategy training for 6 months (MoCA ≥o26). Comparisons of changes of fasting plasma glucose (a), hemoglobin A1c (b), total cholesterol (c), triglyceride (d), low density lipoprotein cholesterol (e), and high‐density lipoprotein cholesterol (f) before and after intervention between the patients with MoCA ≥o26 and patients with MoCA < 26 at the end points. Values were expressed as median (interquartile range)**. *p*** Values were calculated from Mann–Whitney test.

This study also analyzed changes in glucose and lipid metabolism before and after intervention in patients with different levels of cognitive function. After 6 months of intervention among 113 patients with T2DM and cognitive impairment, 37 cases (32.74%) had normal cognitive function (MoCA total score ≥26 points). We compared the changes in glucose and lipid metabolism before and after the intervention between 37 patients whose cognitive function had returned to normal and 76 patients who had mostly improved but had not yet returned to normal. The FPG (*p* = .023), HbA1c (*p* = .012), TC (*p* = .015), TG (*p* = .005), and LDL‐C (*p* = .034) levels were significantly lower in patients with normal cognitive function than in patients with abnormal cognitive function (Figure 4a–e). Consistently, the HDL‐C levels (*p* = .022) were significantly upregulated in the patients with normal cognitive function compared with in patients with abnormal cognitive function (Figure 4f). The results showed that the patients whose cognitive function returned to normal had significantly improved glucose and lipid metabolism indicators before and after the intervention, and there were significant differences.

## 4 DISCUSSION

T2DM is closely related to the occurrence of cognitive dysfunction (Callisaya et al., 2019). With the progression of the disease, patients may show learning disabilities, memory loss, and even dementia in severe cases, causing a heavy burden to the patient's family and the society (Callisaya et al., 2019). The specific mechanism of memory impairment caused by T2DM is not very clear, some studies believe that it is related to hippocampal white matter degeneration (the patient's hippocampus is significantly atrophied) (Gao et al., 2017; Jin et al., 2018). Therefore, active intervention is of great significance to delay or even reduce the occurrence of dementia. At present, there is no special and effective intervention for such patients, and drug treatment is still the mainstay, and the effect is not satisfactory. Studies have shown that exercise not only helps to control blood sugar but also improves cognitive function (Lista & Sorrentino, 2010) and reduces the risk of cognitive impairment (Frederiksen et al., 2015), which is of great significance to patients with T2DM and cognitive impairment. In addition, in recent years, foreign scholars advocate the use of memory strategy training, but its effect lacks sufficient evidence‐based basis, and there is no relevant research report in China. Our hospital has carried out aerobic exercise combined with memory strategy training to provide nursing intervention for patients with T2DM and cognitive impairment for many years. This study retrospectively analyzed the improvement effect of aerobic exercise combined with memory strategy training on cognitive dysfunction and glucose/lipid metabolism in patients with T2DM and cognitive dysfunction.

Shih et al. (2018) conducted a 10‐year follow‐up of 1438 cognitively normal Mexican–American older adults and found that physical activity could reduce the risk of cognitive impairment in T2DM patients. Song et al. (2018) conducted a meta‐analysis of 10 studies, and the results showed that all kinds of exercise improved the cognitive function of patients with mild cognitive impairment, and aerobic exercise improved the cognitive function more significantly. However, since studies have not comprehensively compared the effects of different exercises on blood glucose and cognitive function in patients, the optimal type, intensity, duration, and frequency of exercise in patients with T2DM and mild cognitive impairment have not been determined. In addition, Hampstead et al. (2020) performed memory intervention on elderly patients with amnestic mild cognitive impairment, including memory strategies, word interval retrieval training, and so forth. After 6 weeks of intervention, it was found that the short‐term and long‐term recall of words in the patients had significantly improved, and functional magnetic resonance scans of the brain showed that the electrical activity of the temporal lobe, frontal lobe, and parietal lobe was enhanced (Hampstead et al., 2020). Therefore, it is speculated that memory training can effectively activate the brain network of memory‐related regions. The scores of MoCA and ADL were significantly upregulated, and the scores of DSQL were significantly reduced after the 6‐month intervention in T2DM patients. This study found that the scores of MoCA and ADL were significantly upregulated, and the scores of DSQL were significantly reduced after the 6‐month intervention in T2DM patients. Consistent with previous studies, aerobic exercise combined with memory strategy training can effectively improve cognitive function and quality of life in T2DM patients with cognitive impairment.

Previous studies have reported that abnormal blood lipid levels and the resulting increase in ceramides are one of the risk factors for cognitive impairment in patients with T2DM (Holland et al., 2011). Ceramides block the normal conduction of the insulin signaling pathway by increasing the secretion of pro‐inflammatory cytokines, resulting in the body being in a state of insulin resistance (de la Monte, 2012). The peripherally synthesized ceramides pass through the blood–brain barrier and further exert their pro‐inflammatory and insulin resistance effects in the central nervous system, thereby causing abnormalities in neurodegenerative‐related cell signaling pathways (de la Monte, 2012). In addition, ceramides activate stress‐related signaling pathways and lead to Aβ neurofibrillary tangles by altering the lipid raft microenvironment of cell membranes (De Felice & Ferreira, 2014). Previous animal experiments have verified that the content of ceramides in the blood circulation of high‐fat‐fed rats increases, which is accompanied by mild neurodegeneration (Lyn‐Cook et al., 2009). In another animal experiment, intraperitoneal injection of ceramides into rats resulted in the inhibition of insulin receptor substrate‐1 and Akt signaling pathways in the central nervous system, resulting in impaired neurocognitive function (de la Monte et al., 2010). It can be seen that the increase of ceramides caused by dyslipidemia is one of the important risk factors for impairing cognitive function, and corresponding intervention can be used as a preventive measure to delay cognitive decline or even improve cognitive function. In recent years, a study suggested that 16 weeks of resistance exercise combined with dietary adjustment improves various blood lipid indicators in elderly obese patients, including TC and TG levels (Amamou et al., 2017).

In our study, we observed significant improvements in the levels of FPG, HbA1c, and TG in T2DM patients with cognitive impairment following intervention. Additionally, there was a notable decrease in LDL‐C levels and an increase in HDL‐C levels. Compared to patients with abnormal cognitive function, those with normal cognitive function had significantly lower levels of FPG, HbA1c, TC, TG, and LDL‐C, and higher levels of HDL‐C. These results suggest that combining aerobic exercise with memory strategy training can effectively improve glucose and lipid metabolism in T2DM patients with cognitive impairment, which is closely linked to the recovery of cognitive function. This study emphasizes the importance of addressing cognitive dysfunction in T2DM patients, as it is a common complication that significantly impacts their quality of life and overall well‐being. By incorporating aerobic exercise and memory strategy training, our study demonstrates a potential approach to improve cognitive function in T2DM patients. The findings of this study may have implications for improving the quality of life of T2DM patients and reducing the burden on patients, families, and society. Cognitive impairment in T2DM patients can significantly impact their ability to perform daily activities and their overall quality of life.

Although this study provides valuable insights, it is not without its limitations. The research design only includes pre‐ and postexercise control data for the subjects, and lacks a control group that did not receive any intervention. The evaluation of cognitive function was based solely on the MoCA scale, without the use of imaging techniques such as magnetic resonance imaging of the central nervous system. This may limit the accuracy of cognitive impairment diagnosis and the ability to identify causative factors. Additionally, the MoCA scale itself may have learning and memory effects, which could bias the results. The analysis also included a limited number of clinical observation indicators and did not include follow‐up measures, such as levels of inflammatory factors, oxidative stress indicators, insulin resistance index, and ceramides. As such, the preliminary conclusions of this study require further validation through basic experiments and clinical trials.

## 5 CONCLUSION

In conclusion, our study showed that aerobic exercise combined with memory strategy training effectively improved the memory and cognitive function in patients with T2DM. When comparing patients with different levels of cognitive function, those whose cognitive function returned to normal after the intervention exhibited more significant improvements in glucose and lipid metabolism markers than those patients whose cognitive function improved but did not reach normal levels.

## AUTHOR CONTRIBUTIONS

N.Z, X.Y, Y.W, X.C and Z.W conducted the experiments, collected and analyzed the data, wrote the manuscript. X.Y conceived and supervised the study.

## CONFLICT OF INTEREST STATEMENT

The authors declare that there are no conflicts of interest that could be perceived as prejudicing the impartiality of the research reported.

## FUNDING INFORMATION

This research received no specific grant from any funding agency.

### PEER REVIEW

The peer review history for this article is available at https://publons.com/publon/10.1002/brb3.3234.

## Data Availability

The data could be obtained upon reasonable request to the corresponding author.
